# POEMS Syndrome Masquerading as Metastatic Prostate Cancer Based on PSMA Avid Lesions

**DOI:** 10.1155/crh/4556395

**Published:** 2025-09-03

**Authors:** Pierre Rodriguez Alarcon, Dawid Mehlich, Fidai Shiraz, Matias Sanchez

**Affiliations:** ^1^Department of Hematology and Oncology, John H. Stroger, Jr. Hospital of Cook County, Chicago, Illinois, USA; ^2^Department of Molecular Onco Signalling, International Institute of Molecular Mechanisms and Machines, Polish Academy of Sciences, Warsaw, Poland; ^3^Department of Pathology, John H. Stroger, Jr. Hospital of Cook County, Chicago, Illinois, USA; ^4^Department of Hematology, University of Illinois Health, Chicago, Illinois, USA

## Abstract

Polyneuropathy, organomegaly, endocrinopathy, monoclonal gammopathy, and skin change (POEMS) syndrome is a multisystem disorder, and it is often misdiagnosed with other entities including chronic inflammatory demyelinating polyneuropathy (CIDP). Here, we present a case of a patient with presumed metastatic prostate cancer due to prostate-specific membrane antigen (PSMA) avid lesions and a history of neuropathy not responding to conventional treatment for CIDP. His physical exam findings, in addition to an appropriate workup, led to a diagnosis of POEMS syndrome. This case highlights the importance of a high index of clinical suspicion, even when imaging suggests otherwise.

## 1. Introduction

Polyneuropathy, organomegaly, endocrinopathy, monoclonal gammopathy, and skin change (POEMS) syndrome is a rare paraneoplastic condition associated with plasma cell neoplasm. It is diagnosed through a combination of clinical and laboratory findings, including two mandatory criteria—polyneuropathy and a monoclonal plasma cell disorder—along with at least one additional major and one minor criterion (Table 1) [1]. Due to its rarity, diverse symptoms, and involvement of multiple organs, POEMS syndrome is often difficult to diagnose, leading to delays in recognition and treatment.

Here, we present a case of a patient diagnosed with POEMS syndrome and synchronous prostate cancer. This report highlights the relevant diagnostic challenges associated with POEMS syndrome, particularly in patients with a concurrent history of other malignancies.

## 2. Case Presentation

We present the case of a 62-year-old gentleman with past medical history of chronic venous insufficiency, diabetes, and chronic bilateral weakness in hands and legs with associated distal sensory loss in feet and hands that had been previously attributed to CIDP, diagnosed in 2021. EMG obtained at diagnosis was abnormal with electrophysiologic evidence of an acquired demyelinating polyneuropathy with secondary axon loss. Ultrasound impression was also abnormal, with the UPSS-A score of 11, indicating enlargement of several nerves in proximal segments, suggesting a diagnosis of CIDP. The patient had been following up with neurology and had tried intermittent courses of steroids and IVIG with little to no improvement in neurological defects.

**Figure 1:**
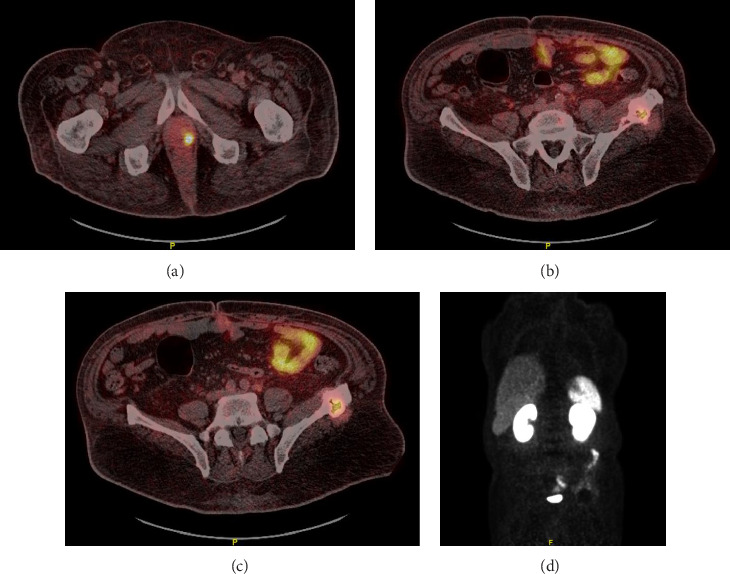
PSMA PET-CT imaging findings. (a) PSMA avid lesion in the posterior aspect of the left prostate lobe; (b–d) PSMA avid left retroperitoneal and left iliac chain nodes and PSMA avid lesion within the left iliac bone extending from the iliac wing down to the anterior acetabulum.

The patient was also diagnosed with prostate cancer (Gleason score 4 + 3, iPSA 6.3) in August 2023. A bone scan was done initially showing mildly increased uptake in bilateral shoulders, wrists, knees, ankles, and feet which were most consistent with degenerative disease but no evidence of metastatic disease. Staging computed tomography (CT) of the abdomen and pelvis was obtained and showed hepatomegaly and enlarged pelvic lymph nodes with prominent retroperitoneal lymph nodes and nonspecific mixed lytic and sclerotic appearance of the left iliac crest without significant corresponding FDG update on comparison bone scan. Due to concern for metastatic disease, a PSMA scan was obtained which showed an intense PSMA avid lesion in the posterior aspect of the left prostate lobe concerning for site of primary prostatic malignancy (Figure 1(a)), a mildly PSMA avid subcentimeter left retroperitoneal and left iliac chain nodes concerning for nodal metastasis and a large continuous mixed lytic/sclerotic PSMA avid lesion within the left iliac bone extending from the iliac wing down to the anterior acetabulum (Figures 1(b), 1(c), and 1(d)). His PSA was elevated to 4.99 ng/mL. The patient was referred to our Oncology clinic for consideration of systemic treatment of metastatic prostate cancer.

**Figure 2:**
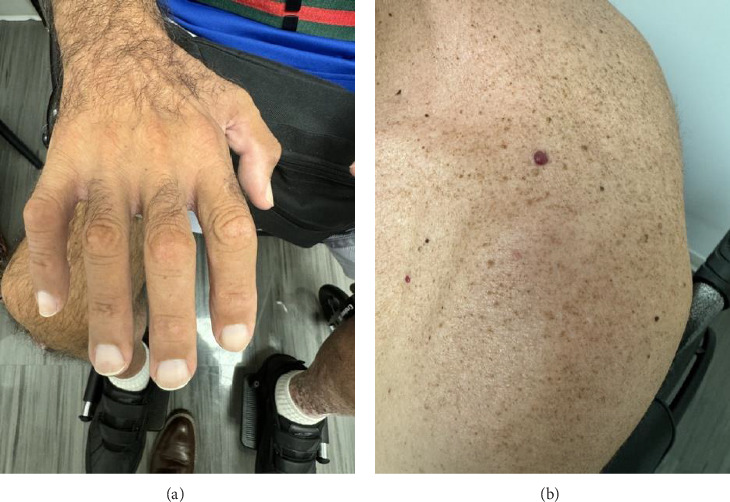
Physical examination findings: (a) white discoloration of the nails (leukonychia); (b) skin hemangioma on the left shoulder.

Physical examination was remarkable for bilateral lower extremity edema and hyperpigmentation that had been attributed to chronic venous insufficiency, hypertrichosis, hemangiomata in the thorax, arms and scalp, and white nails that the patient's partner had noticed over the course of the last few months (Figure 2). At this time, metastatic prostate cancer felt to be less likely given prior history and exam findings.

Blood workup for paraproteinemia and biopsy of the sclerotic bone lesion as well as retroperitoneal lymphadenopathy were obtained to rule out a monoclonal gammopathy as a culprit for the patient's clinical presentation. Serum protein electrophoresis (SPEP) with immunofixation was positive for a monoclonal IgG lambda with normal kappa (14.7 mg/L-Normal: 3.3–19.4 mg/L) and lambda (18.5-Normal: 5.7–26.3 mg/L) serum light chains. Additional blood workup included vascular endothelial growth factor (VEGF) levels, quantitative immunoglobulins, and a complete hormonal profile including luteinizing hormone (LH), follicular stimulant hormone (FSH), prolactin, insulin-like growth factor 1 (IGF-1), parathyroid hormone (PTH), and thyroid stimulant hormone (TSH) (Table 2).

**Figure 3:**
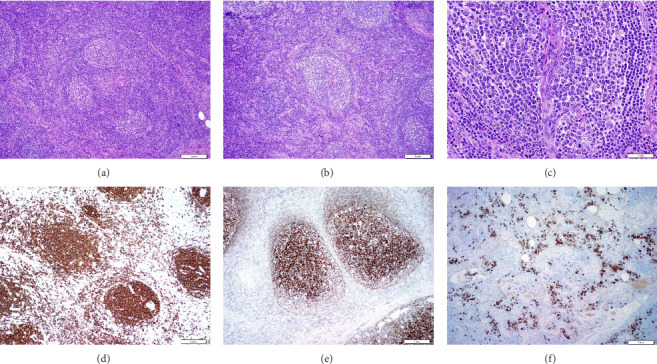
Histopathology.

Biopsy of the left iliac bone was negative for carcinoma (immunohistochemical stains such as pankeratin, NKX3.a, PSA, and PSAP were included and negative); however, the foci of plasma cell clusters (CD 138 positive) with slight lambda light chain excess were found. This finding supported our suspicion of POEMS syndrome. Hence, a bone marrow biopsy was obtained, showing a normocellular to mildly hypercellular bone marrow (30%–50% cellularity) with progressive trilineage hematopoiesis and 5%–7% plasma cells with mild lambda light chain excess (in a background of polyclonal kappa positive plasma cells), negative for metastatic prostate adenocarcinoma and negative for amyloid deposition. No circulating plasma cells or rouleaux formation was seen in peripheral blood. No high-risk cytogenetics was identified, and the patient had an apparent normal karyotype. Interventional radiology (IR)–guided biopsy of a left inguinal lymph node (core biopsy was obtained) was positive for changes most consistent with Castleman disease, negative for HHV-8 negative and involvement by metastatic prostate carcinoma or amyloid deposition (Congo red stain negative) (Figure 3).

With all this information, a diagnosis of POEMS syndrome was made. The patient was referred to radiation oncology for evaluation for pelvic external beam radiation therapy (EBRT) for localized prostate cancer. He started daratumumab, pomalidomide, and dexamethasone-based regimen. He completed RT to the prostate and achieved improvement of neuropathy and weakness in his extremities.

## 3. Discussion

Despite significant progress in defining distinctive clinical and laboratory criteria for POEMS, making the diagnosis remains challenging and requires high clinical suspicion, detailed history, and appropriate testing. As illustrated in this case, polyneuropathy is often the initial manifestation of the disease. The electrophysiological findings in patients with POEMS reflect nerve demyelination, and therefore, the disorder is commonly misdiagnosed as CIDP. In retrospective analyses, 50%–60% of patients with POEMS syndrome were initially diagnosed with CIDP, and most of them fulfilled the electrodiagnostic criteria for definitive CIDP [2, 3]. Clinically, patients with POEMS neuropathy were found to be more likely to have severe leg pain, muscle atrophy, and distal dominant muscle weakness compared to CIDP [2]. Several observational studies reported that POEMS neuropathy can be distinguished from CIDP by distinct features on nerve conduction studies, electromyography, high-resolution nerve ultrasound, and nerve biopsy [1]. While these findings provide valuable insights, their clinical utility may be limited in cases where other radiological and laboratory results support the diagnosis of POEMS. Plasma or serum VEGF levels are commonly used to differentiate POEMS from CIDP and other immune-mediated neuropathies [4]. The recently published cost-effectiveness analysis based on clinical data of 100 POEMS patients treated at the University College London Hospital suggested that early VEGF testing in patients presenting with symptoms of inflammatory neuropathies may prevent diagnostic delay, disease progression, and significant healthcare costs associated with misdiagnosis of POEMS [3]. Thrombocytosis in routine labs was significantly more common in patients with POEMS than CIDP (53.7% vs. 1.5%) and may aid in distinguishing between these two entities [4]. Radiographic assessment of bones in patients with POEMS syndrome typically reveals osteosclerotic lesions, while mixed or lytic lesions are less commonly detected. CT imaging is highly sensitive for detecting bone lesions associated with POEMS syndrome and plays a role in evaluating additional diagnostic criteria, such as volume overload and organomegaly [5, 6].

The co-occurrence of POEMS syndrome with other solid malignancies is extremely rare, with only a few cases described to date, including breast cancer, prostate cancer, and gallbladder adenocarcinoma [7–9]. In several large retrospective studies, synchronous malignancies were not reported or systematically analyzed, reflecting the rarity of this presentation [10, 11]. Consequently, there is no substantial evidence that specific types of cancer are associated with POEMS syndrome. As illustrated here, in rare cases of the coexistence of POEMS syndrome with other solid tumors, the imaging findings may appear ambiguous and difficult to interpret. In these patients, manifestations of POEMS such as bone lesions, pleural effusion, ascites, and lymphadenopathy may be attributed to metastatic disease. This highlights the unique diagnostic challenges and underscores the need for heightened clinical vigilance.

PSMA is a Type II transmembrane glycoprotein that is commonly overexpressed in prostate cancer cells and has become an important target molecule for imaging in clinical practice [12]. On December 1, 2020, the U.S. Food and Drug Administration approved Gallium 68 PSMA-11 (Ga 69 PSMA-11) as the first drug for positron emission tomography (PET) imaging of PSMA-positive lesions in men with suspected metastatic disease or biochemical recurrence based on prostate-specific antigen elevation following prostate surgery or RT. This was followed by the approval of a second agent, Piflufolastat F 18 (Pylarify) on May 27, 2021, and a third agent, Flotufolastat F18 (Posluma) on May 30, 2023. These PSMA-targeted molecules bind to and are internalized by PSMA-expressing cells such as prostate cancer cells. This process results in the tracer accumulation in the tumor cells over time, thereby enabling a specific and sensitive diagnosis of advanced disease. Of note, PSMA is not exclusively expressed by prostate cells. Its expression has been documented in extraprostatic tissues, including the brain, salivary glands, and small intestine [13]. Additionally, it is present in endothelial cells of capillary beds in various malignancies where angiogenesis is important for tumor development and progression, such as colon adenocarcinoma and renal cell carcinoma, among others [14].

The pathogenesis of POEMS syndrome is, to date, not well understood; however, elevated VEGF levels correlate with disease activity and is a marker often used to follow and assess response to treatment [15]. There is evidence supporting PSMA expression in tumor-associated neo-vasculature [16]; however, its role in plasma cell disorders is less understood. There are case reports with PSMA avid lesions in patients with multiple myeloma, suggesting that tumor angiogenesis is the mechanism associated with increased uptake of Gallium 68 PSMA-11 [17]. Whether or not the same mechanism associated with POEMS syndrome is still unknown.

In summary, we describe an unusual presentation of POEMS syndrome and synchronous prostate cancer, emphasizing the diagnostic challenges and importance of careful imaging interpretation in such cases. Notably, PSMA uptake, although highly specific for prostate cancer, can also be seen in POEMS syndrome. This underscores the importance of cautious interpretation of PSMA PET findings in complex clinical situations.

## Figures and Tables

**Table 1:** Criteria for the diagnosis of POEMS syndrome.

Mandatory major criteria	1. Polyneuropathy (typically demyelinating)
	2. Monoclonal plasma cell-proliferative disorder (almost always lambda—*λ*)

Other major criteria (one required)	3. Castleman disease
	4. Sclerotic bone lesions
	5. VEGF elevation

Minor criteria	6. Organomegaly (splenomegaly, hepatomegaly, or lymphadenopathy)
	7. Extravascular volume overload (edema, pleural effusion, or ascites)
	8. Endocrinopathy (adrenal, thyroid, pituitary, gonadal, parathyroid, pancreatic)
	9. Skin changes (hyperpigmentation, hypertrichosis, glomeruloid hemangiomata, plethora, acrocyanosis, flushing, white nails)
	10. Papilledema
	11. Thrombocytosis/polycythemia

Other symptoms and signs	12. Clubbing, weight loss, hyperhidrosis, pulmonary hypertension/restrictive lung disease, thrombotic diatheses, diarrhea, low vitamin B12 values

**Table 2:** Laboratory workup.

Complete blood count (CBC)	Hemoglobin: 15.3 g/dL (normal: 12.9–16.8 g/dL)
	Hematocrit: 45.7% (normal: 38.1%–49%)
	WBC: 9.5 k/μL (normal: 4.4–10.6 k/μL)
	Platelets: 586 k/μL (normal: 161–369 k/μL)
Basic metabolic profile	
VEGF	4075 pg/mL (normal: 31–86 pg/mL)
FSH	5.24 mIU/mL (normal: 1.27–19.26 mIU/mL)
LH	7.12 mIU/mL (normal: 1.24–8.62 mIU/mL)
Prolactin	7.63 ng/mL (normal: 2.64–13.13 ng/mL)
PTH	24.39 pg/mL (normal: 12–88 pg/mL)
Cortisol	12.42 μg/dL
IGF-1	181 ng/mL (normal: 41–279 ng/mL)
TSH	1.59 μIU/mL (normal: 0.34–5.60 μIU/mL)
Quantitative immunoglobulins	IgG: 1160 mg/dL (normal: 694–1618 mg/dL)
	IgA: 329 mg/dL (normal: 68–378 mg/dL)
	IgM: 41 mg/dL (normal: 77–220 mg/dL)

## Data Availability

All data underlying the findings of this case report are included in the article. Additional data are available from the corresponding author upon request.
